# Pathogenic Role of mTOR Signaling in Cardiometabolic Disease: Implications for Heart, Liver, and Kidney Dysfunction

**DOI:** 10.33549/physiolres.935677

**Published:** 2025-12-01

**Authors:** Mahak ARORA, Josef ZICHA, Ivana VANĚČKOVÁ

**Affiliations:** 1 Department of Experimental Hypertension, Institute of Physiology, Czech Academy of Sciences, Prague, Czech Republic

**Keywords:** Cardiometabolic disease, mTOR, Dyslipidemia, Salt sensitive, Hypertension

## Abstract

Cardiometabolic diseases (CMDs), which include hypertension, atherosclerosis, chronic kidney disease, type 2 diabetes mellitus (T2DM), metabolic syndrome, and obesity, significantly affect the heart, liver, and kidneys. A key player in the pathogenesis of these diseases is the serine-threonine kinase enzyme mTOR (mammalian target of rapamycin), which affects cellular metabolic processes through its signaling. mTOR is composed of two separate complexes: mTORC1 and mTORC2. Both complexes are essential for cardiac development and pathological stress responses. Constant activation of mTORC1 can be harmful, contributing to cardiac hypertrophy and remodeling, which can lead to heart failure. Conversely, mTORC2 supports the survival and function of cardiomyocytes during stressful situations. In the liver, mTOR signaling plays a crucial role in lipid metabolism and insulin sensitivity, both of which are affected by diet. Activation of mTORC1 in hepatocytes can cause hepatic steatosis, dyslipidemia, and insulin resistance, which are characteristics of metabolic dysfunction and type 2 diabetes mellitus (T2DM). Conversely, mTORC2 protects against steatohepatitis. Reducing mTORC1 activity in the liver improves these metabolic disturbances. Altered mTOR signaling may result from abnormal feeding states, which affect the metabolic and physiological functions of the liver and kidneys. In diabetic nephropathy, overstimulation of mTORC1 in the kidneys leads to hypertrophy, proteinuria, and eventual loss of renal function. Meanwhile, mTORC2 participates in renal ion transport. Treatment with mTOR inhibitors has ameliorated renal dysfunction in preclinical models of diabetic kidney dysfunction and the Dahl S model of salt hypertension. This review emphasizes the critical role of mTOR in the pathophysiology of cardiometabolic diseases in major organs and models. Targeting mTOR signaling pathways is a promising approach to mitigate the adverse effects of CMD on the heart, liver, and kidneys.

## Introduction

Cardiometabolic disorders (CMDs) are a group of interconnected conditions that significantly increase the risk of cardiovascular disease and metabolic abnormalities. Examples of these disorders include hypertension, type 2 diabetes, dyslipidemia (abnormal cholesterol and triglyceride levels), and obesity. Insulin resistance is often an underlying factor that contributes to the development of these conditions. Each disorder presents unique health challenges. For example, hypertension can lead to heart or kidney failure and stroke, while type 2 diabetes is associated with neuropathy and kidney damage. Dyslipidemia accelerates atherosclerosis, and obesity increases systemic inflammation and metabolic stress. These conditions often coexist, amplifying their negative effects on health and necessitating integrated prevention and management strategies. Lifestyle interventions, such as a healthy diet, regular physical activity, and weight management, are fundamental in mitigating the risk of cardiometabolic disorders and are often combined with pharmacological interventions to control specific risk factors [1,2].

## Epidemiology

The epidemiological prevalence of cardiometabolic disorders has reached alarming levels globally. Lifestyle changes, urbanization, and an aging population drive this increase. Conditions such as obesity, type 2 diabetes, hypertension, and dyslipidemia are becoming increasingly common, particularly in low- and middle-income countries that are experiencing rapid socioeconomic transitions [3]. Hypertension is a major contributor to premature death globally. For instance, the World Health Organization (WHO) estimates that 1.28 billion adults worldwide have hypertension. WHO has reported recently a striking surge in the WHO Asia Pacific/Southeast Asia region [4]. The prevalence of obesity has tripled since 1975, affecting over 650 million adults and significantly contributing to metabolic syndrome [5]. Type 2 diabetes affects approximately 537 million adults globally, according to the International Diabetes Federation (IDF), with projections suggesting this figure could rise to 643 million by 2030 [6]. According to the International Diabetes Federation (IDF), type 2 diabetes affects approximately 537 million adults globally [7]. Dyslipidemia is a critical risk factor that is often underdiagnosed. Studies show that its prevalence rate is as high as 40 % in some populations, particularly in developed regions. Metabolic syndrome, which is defined as a group of these disorders, affects up to 25 % of the global adult population, which underscores its widespread impact [8].

## Pathogenesis of Human Cardiometabolic Disorders

**Fig. 1:**
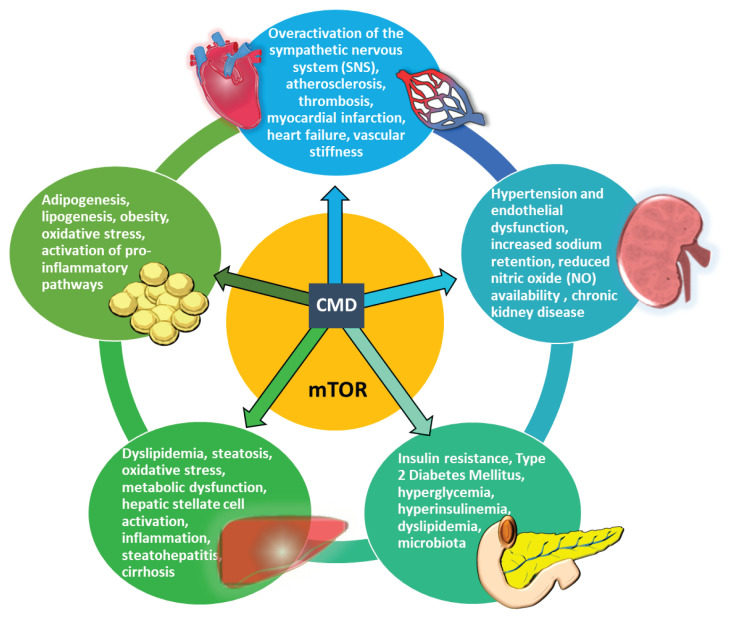
Vicious cycle of cardiometabolic diseases.

Cardiometabolic diseases, including obesity, insulin resistance, type 2 diabetes mellitus (T2DM), hypertension, dyslipidemia, and cardiovascular diseases, are deeply interconnected through overlapping pathophysiological mechanisms (Fig. 1). These diseases do not occur in isolation, but rather, they share common risk factors and pathological pathways that exacerbate each other. It is important to note how they are interlinked. Obesity, a central driver, may result in adipose tissue dysfunction, leading to chronic, low-grade inflammation due to the secretion of pro-inflammatory cytokines, such as Tumor Necrosis Factor alpha (TNF-α) and interleukin-6 (IL-6). Insulin resistance occurs due to elevated free fatty acids and a disrupted balance of adipokines (low adiponectin and high leptin levels) [9]. Ectopic fat accumulation in the liver, muscle, and pancreas leads to metabolic dysfunction. Therefore, obesity increases the risk of insulin resistance, type 2 diabetes mellitus, hypertension, and cardiovascular disease by promoting systemic inflammation, oxidative stress, and endothelial dysfunction [10]. Chronic insulin resistance in muscle, liver, and adipose tissue can lead to the development of type 2 diabetes mellitus. Furthermore, hyperglycemia occurs due to reduced cellular glucose uptake. Hyperinsulinemia occurs due to a compensatory increase in insulin secretion. Dyslipidemia occurs due to increased triglycerides, decreased high-density lipoprotein (HDL), and small, dense low-density lipoprotein (LDL) formation. Endothelial dysfunction occurs due to impaired nitric oxide production. Hyperglycemia and hyperinsulinemia contribute to vascular damage and accelerate atherosclerosis, thereby increasing the risk of cardiovascular disease. Conversely, dyslipidemia and oxidative stress exacerbate metabolic syndrome, which encompasses hypertension and central obesity [11]. Insulin resistance leads to excessive hepatic fat accumulation by increasing lipogenesis and decreasing fat oxidation. Dyslipidemia worsens hepatic steatosis, leading to lipid toxicity. Obesity-induced inflammation results in the chronic activation of proinflammatory pathways, accelerating the progression of metabolic dysfunction-associated steatotic liver disease (MASLD) [12]. Hyperinsulinemia may promote hypertension via activation of the sympathetic nervous system (SNS), although direct experimental evidence remains limited. Reduced nitric oxide (NO) availability [13] leads to increased vascular resistance and SNS overactivation, which causes an increase in blood pressure [14]. Insulin normally promotes renal sodium excretion, but this function is impaired, leading to sodium retention, body fluid expansion, and increased blood pressure [15]. Hypertension is a major risk factor for stroke, heart failure, and renal disease [16]. Endothelial dysfunction exacerbates atherosclerosis, further increasing cardiovascular disease risk. Hypertension and endothelial dysfunction also impair hepatic microcirculation, exacerbating ischemic injury in the liver [17].

Furthermore, there are numerous potential pathways linking gut microbiota to an individual’s cardiometabolic health that have not been well explored. Commensal gut microbes can influence CMD pathogenesis by modulating nutrient and energy availability, among other mechanisms. Another shows that some gut bacteria, such as Lactobacillus murinus, inhibit the activation of pathogenic TH17 cells. However, these bacteria are depleted by high-salt intake, which may result in an increased risk of hypertension and inflammatory disorders [18]. In some cases, gut microbe-derived molecules, such as bile acids, affect T-cell activity, revealing the intricate relationship between gut microbiota and the innate immune system. Bacterial products like lipopolysaccharide enter through the portal vein, activating systemic immune responses and leading to a proinflammatory state with altered lipopolysaccharide responsiveness and an increased risk of cardiometabolic disease, heart failure, and adverse outcomes [19]. Metabolites produced by the host and microbiota, such as trimethylamine N-oxide (TMAO) and phenyl-acetylglutamine, are associated with an increased risk of atherosclerosis, thrombosis, and heart failure, respectively [20].

Dyslipidemia and atherosclerosis may result from insulin resistance, which increases the production of very-low-density lipoprotein (VLDL) and small, dense LDL particles that are more susceptible to oxidation. This leads to low HDL levels and reduced cholesterol clearance [21]. Atherogenic dyslipidemia promotes plaque formation, leading to myocardial infarction, stroke, and peripheral artery disease [22]. Chronic inflammation from dyslipidemia and type 2 diabetes mellitus further accelerates plaque instability, increasing cardiovascular disease risk [23]. Chronic inflammation and oxidative stress in CMD may increase the incidence of cardiovascular disorders. Persistent inflammation in obesity, type 2 diabetes mellitus, and dyslipidemia activates the NLRP3 (nucleotide-binding domain-leucine-rich repeat-containing protein 3) inflammasome, which promotes systemic inflammation, increased oxidative stress, and vascular damage. Enhanced foam cell formation worsens atherosclerosis. Additionally, activation of the renin-angiotensin system (RAS) promotes fibrosis through angiotensin II-mediated stellate cell activation [24]. Chronic inflammation and oxidative stress drive vascular aging, increasing susceptibility to hypertension, atherosclerosis, and heart failure. In the liver, hyperinsulinemia promotes hepatic fibrosis by activating hepatic stellate cells, which produce excess collagen and the extracellular storage matrix. Chronic inflammation by chemokines like TNF-α and IL-6 further stimulates fibrogenesis, increasing the risk of cirrhosis [25]. Persistent insulin resistance and chronic inflammation increase hepatic oncogenic signaling (PI3K-AKT and JNK pathways) and significantly increase the risk of hepatocellular carcinoma development [26].

Therefore, it persists as a vicious cycle of cardiometabolic diseases. These factors reinforce each other, creating a self-perpetuating cycle of cardiovascular events accompanied by metabolic dysfunctions. Inflammation and oxidative stress worsen insulin resistance and endothelial dysfunction. Metabolic dysfunction can lead to organ damage, such as kidney or liver disease and heart failure, which can further amplify the above cycle. Cardiometabolic diseases are interconnected through metabolic, inflammatory, and vascular pathways. A holistic approach is required to address these diseases, including lifestyle changes (e.g., diet and exercise), pharmacotherapy (e.g., anti-diabetic, lipid-lowering, and antihypertensive drugs), and emerging therapies that target gut microbiota and inflammation.

In humans, we have particular biomarkers such as fasting plasma glucose (FPG) and glycated hemoglobin A1c (HbA1c) measurements to diagnose the above parameters of CMD, including insulin resistance and type 2 diabetes. FPG measures actual glucose levels and indicates impaired fasting glucose (IFG), whereas HbA1c reflects average glucose levels over two to three months and is a reliable marker for chronic hyperglycemia in humans [27]. Another validated method to assess insulin resistance is the homeostatic model assessment for insulin resistance (HOMA-IR) [28]. Serum or tissue values of low-density lipoprotein cholesterol (LDL-C) and high-density lipoprotein cholesterol (HDL-C), along with triglycerides (TG) and non-HDL cholesterol, act as biomarkers for dyslipidemia and atherosclerosis. These values can also be important diagnostic parameters for cardiometabolic syndrome [29]. Markers of inflammation and endothelial dysfunction include C-reactive protein (CRP) and high-sensitivity CRP (hs-CRP). Elevated CRP levels are strongly linked to an increased risk of myocardial infarction and stroke [30]. Measurements of cytokines, such as IL-6 and TNF-α, can predict systemic inflammation [31]. B-type natriuretic peptide (BNP) and N-terminal pro-BNP (NT-proBNP) are cardiac stress markers that help diagnose heart failure and left ventricular dysfunction [32]. Pulse wave velocity (PWV) is a measure of arterial stiffness and an early indicator of hypertension and atherosclerosis. Carotid intima-media thickness (CIMT) evaluates the amount of atherosclerotic plaque in the carotid arteries [33]. Non-invasive techniques for assessing organs include imaging techniques such as ultrasound, computed tomography, magnetic resonance imaging, and electrocardiography for the heart, as well as transient elastography. Liver function tests may include invasive techniques such as a rarely used liver biopsy with histopathological scoring for fatty liver or fibrosis. Beyond this, serum estimations of liver biochemical markers, such as aspartate aminotransferase (AST), alanine aminotransferase (ALT), alkaline phosphatase (ALP), gamma-glutamyl transpeptidase (GGT), bilirubin, as well as the serum lipid profiling, triglycerides (TG), and albumin, may be considered biomarkers for cardiometabolic dysfunction. Inflammatory serum markers include tumor necrosis factor-alpha (TNF-α), adiponectin, C-reactive protein (CRP), and interleukin-6 (IL-6). Cytokeratin 18 is a marker of apoptosis in liver injury, and plasma homocysteine levels can distinguish metabolic dysfunction-associated steatohepatitis (MASH) from simple steatosis [34]. One newly studied marker is osteopontin, a hepatic stellate cell-activating signaling molecule associated with cell injury and fibrosis. Therefore, plasma osteopontin concentration is a predictive marker of liver fibrosis in various hepatic diseases [35]. Renal biomarkers in cardiometabolic syndrome include the estimation of the glomerular filtration rate (eGFR), serum creatinine, and the albumin-to-creatinine ratio (ACR) [36]. These are predictive markers of chronic kidney disease and cardiovascular mortality [37]. Cystatin C is a more sensitive marker than creatinine for detecting early renal dysfunction [38].

## Experimental Models for Inducing Cardio-metabolic Disease in Rodents

Various rodent models have been developed to elucidate the pathophysiology of CMD and guide therapeutic strategies (Table 1). However, no single rodent model can display the entire spectrum of the disease and its associated metabolic features, as they can only display particular characteristics. The most commonly used dietary rodent models for CMD are based on a High-Fat Diet (HFD) or a High-Fat/High-Cholesterol diet (HFC). These include a High-Fat/High-Cholesterol diet (HFC) [39], a high fat/high fructose diet (ALIOS) [40], a high fat/high cholesterol/high fructose diet (AMLN), and a high sucrose and fructose diet (HSF) [41]. There are also chemical models, such as the streptozotocin-induced diabetes model [42] and the dexamethasone-induced metabolic syndrome model [43]. Some genetic models of metabolic dysfunction-associated steatohepatitis (MASH) are also available, such as Zucker fatty rats and Zucker diabetic fatty rats [44]. However, various studies suggest that dietary models are more advantageous than genetic and chemical models because they better mimic the sedentary lifestyle and conditions that lead to the progression of cardiometabolic disease in humans.

Recent studies suggest that a high-salt diet in Dahl rats can effectively model cardiometabolic dysfunction in association with dietary models. The Dahl salt-sensitive (Dahl-SS) rat is a widely used genetic model for studying hypertension, metabolic syndrome, and cardiovascular disease induced by a high-salt diet (HSD) [45]. High salt intake increases inflammatory cytokines (TNF-α and IL-6), which promote insulin resistance and changes in the lipid profile (elevated triglycerides and LDL and reduced HDL) [46]. This model mimics human salt-sensitive hypertension, an important risk factor for cardiometabolic disorders.

High salt intake leads to cardiovascular remodeling and kidney damage, as evidenced by left ventricular hypertrophy (LVH) resulting from pressure overload, as well as enlarged kidneys [47]. Kidney dysfunction may result from high salt intake, leading to glomerular injury, which can cause proteinuria, kidney fibrosis, and chronic kidney disease [48]. Inflammation and fibrosis worsen cardiometabolic dysfunction. Systemic inflammation is associated with myocardial fibrosis, diastolic dysfunction, and cardiac remodeling, all of which contribute to the progression of cardiometabolic diseases [49]. This model affects the cardiovascular, renal, and metabolic systems, thereby mimicking CMD progression and including hepatic metabolic functions that are not yet fully understood. Emerging studies show that a high-salt diet can adversely affect liver health, particularly in salt-sensitive individuals, such as Dahl salt-sensitive rats. These effects are mediated through mechanisms involving oxidative stress, resulting in cellular damage and fibrosis [50]. A high-salt diet induces significant changes in liver metabolic activities at the transcriptional level even before substantial morphological damage is evident, leading to hepatic inflammation and metabolic abnormalities [51].

## Molecular Therapeutic Targets

Cardiometabolic disorders include hypertension, obesity, type 2 diabetes, dyslipidemia, and cardiovascular disease. Clinically approved pharmacological interventions target metabolic, inflammatory, and cardiovascular pathways to reduce morbidity and mortality. First-line therapies for hypertension and heart failure include antihypertensive agents, such as Angiotensin-Converting Enzyme inhibitors (ACE) inhibitors and Angiotensin Receptor Blockers (ARBs), which reduce cardiovascular events [52,53]. Statins (e.g., atorvastatin and rosuvastatin) remain the cornerstone of lipid management, significantly lowering LDL cholesterol and cardiovascular risk [54]. Metformin improves insulin sensitivity for diabetes management, and newer agents like SGLT2 inhibitors (e.g., empagliflozin) and GLP-1 receptor agonists (e.g., liraglutide) offer cardiovascular and renal benefits beyond glycemic control [55–57]. These evidence-based treatments form an integrated strategy to manage cardiometabolic diseases and improve long-term outcomes.

Cardiometabolic diseases, including hyper-tension, obesity, type 2 diabetes, and dyslipidemia, share common molecular pathways, and mammalian target of rapamycin (mTOR) is a pharmacological target for improving metabolic and cardiovascular outcomes.

## mTOR as a Possible Therapeutic Target for Cardiometabolic Disorders

The mammalian target of rapamycin (mTOR) is a serine/threonine kinase that belongs to the phosphatidylinositol 3-kinase (PI3K) family. mTOR assembles into two distinct multiprotein complexes, mTORC1 and mTORC2, which are characterized by their unique subunit compositions. Within these complexes, mTOR acts as the central catalytic component flanked by specific regulatory proteins: raptor in mTORC1 and rictor in mTORC2 [58]. The protein was first identified as the molecular target of rapamycin, an antifungal compound produced by the bacterium Streptomyces hygroscopicus. Rapamycin binds to the 12-kDa FK506-binding protein (FKBP12), forming a complex that inhibits mTORC1 activity by binding to its FKBP12-rapamycin binding (FRB) domain. While some mTORC1 functions remain unaffected, many of its kinase activities are significantly suppressed by rapamycin. [59].

In hepatic tissue, mTORC1 plays a central role in lipid biosynthesis, particularly through its involvement in insulin signaling. When there is an accumulation of excess triglycerides and carbohydrates in the liver and adipose tissue, insulin binds to its receptor on the cell surface. This initiates a cascade that activates PI3K, which subsequently activates protein kinase B (AKT/PKB). Akt then modulates the expression of genes responsible for glucose uptake, glycolysis, and lipid synthesis. This insulin-dependent activation of Akt further stimulates mTORC1, which drives fatty acid production by activating Sterol Regulatory Element-Binding Proteins (SREBPs), especially SREBP-1c and SREBP-2. These proteins are highly expressed in tissues involved in lipogenesis [60]. Overactivation of this pathway is linked to excessive fat accumulation and the development of hepatic insulin resistance, contributing to cardiometabolic syndrome [61]. Porstmann and his co-workers further support this link by demonstrating that SREBP activation downstream of the PI3K/AKT/mTORC1 axis integrates mitogenic cues with nutrient status to regulate protein and lipid synthesis [62]. Similarly, metformin, an antidiabetic agent known to activate AMPK and inhibit mTOR signaling, reduces lipid accumulation in the liver by suppressing SREBP-1c expression [63].

The anti-obesity effects of rapamycin were studied in C57BL/6J mice fed a high-fat diet (HFD). After 16 weeks of treatment with 2 mg/kg of rapamycin, the mice showed reduced food intake, body weight, and serum leptin and insulin levels compared to untreated HFD controls. These mice also had less epididymal and retroperitoneal fat, smaller adipocytes, and improved fatty liver scores. These results highlight the potential of mTOR inhibitors in treating lipid accumulation and cardiometabolic syndrome [64]. Furthermore, rapamycin treatment alleviated liver cell injury, inflammation, and fibrosis in the early stages of cirrhotic hypertension in bile duct-ligated rats, underscoring its protective role in liver disease [65]. In vivo studies have linked mTOR signaling to the progression of metabolic-associated fatty liver disease (MAFLD). Sprague-Dawley rats fed a high-fat diet for eight or 16 weeks exhibited elevated liver enzymes and progressive steatosis severity. Both groups had significantly higher expression of mTOR, S6K1, IL-1α, IL-6, and TNFα compared to controls. These results suggest that hepatic mTOR may be a promising therapeutic target for treating cardiometabolic disorders [66].

The role of mTORC1 in lipid biosynthesis is well established, but studies on mTORC2 are limited. However, emerging evidence shows that mTORC2 also regulates hepatic lipid metabolism. Hagiwara et al. (2012) found that Rictor knockout mice exhibited reduced SREBP activity and impaired lipogenic gene expression. This suggests that mTORC2 plays a role in regulating glucose and lipid homeostasis through Akt phosphorrylation at Ser473. These results suggest that mTORC2 influences hepatic glucose and lipid metabolism by regulating insulin-Akt phosphorylation at Ser473, thereby controlling metabolic homeostasis overall [67]. While mTORC1 may compensate through Akt overexpression, other studies have reported that loss of mTORC2 shifts brown fat metabolism from lipogenesis to oxidation, thereby protecting against obesity [68]. Reduced mTORC2 activity also leads to decreased mTORC1 activity and SREBP signalling [69]. Since mTORC1 inhibitors, such as rapamycin, are not mTORC2-selective [70], further research is necessary to develop targeted therapies for metabolic diseases. Recently, we demonstrated the beneficial effects of acute and chronic Ku-0063794 (KU) treatment in C57BL/6J mice with steatohepatitis induced by a high-fat diet (HFD). KU effectively improved certain steatohepatitis parameters, including oxidative stress, ATP production, and MASH biochemical markers, without producing side effects in other organs [71].

mTOR participates in regulating renal function by modulating kidney repair and disease progression. In rodent models of chronic kidney disease and diabetic nephropathy, increased mTORC1 activity leads to fibrosis and glomerular hypertrophy. These effects can be mitigated by rapamycin treatment, though it has side effects [72,73]. mTORC2 is essential for regulating potassium homeostasis in the kidney [74,75]. Similarly, a mouse model of polycystic kidney disease demonstrated excessive mTOR activation leading to cyst expansion, which was mitigated by rapamycin [76]. Rictor/mTORC2 signaling activation mediates TGFβ1-induced fibroblast activation and contributes to kidney fibrosis development. Therefore, it could be a crucial target for treating chronic kidney disease [77]. In a mouse model of acute kidney injury, pharmacological inhibition of either mTOR or STAT3 significantly improved renal function and reduced apoptosis and inflammation [78]. DOCA-salt hypertensive rats exhibited elevated blood pressure (BP), increased relative kidney weight, and PI3K/Akt/mTOR pathway activation in kidneys, as evidenced by altered apoptotic and inflammatory markers [79]. Inhibiting mTORC2 and/or NADPH oxidase could also be a treatment option for diabetic kidney disease, as observed in the glomeruli of OVE26 mice in isolation [80,81]. These studies highlight the mTOR pathway as a potential therapeutic target for kidney diseases.

The mTOR pathway plays a crucial role in regulating the cardiovascular system’s function. It is necessary for the cardiovascular development in the embryo after birth and for preserving cardiac homeostasis [82]. Studies of mice with lost mTOR function have shown that adaptive cardiac hypertrophy in response to mechanical overload depends on the activation of mTORC1 [83]. mTORC2 is necessary for appropriate cardiac function and significantly influences the survival of cardiomyocytes under pressure overload conditions. Conversely, partial genetic or pharmacological suppression of mTORC1 reduces heart remodeling and heart failure induced by chronic myocardial infarction and pressure overload. Furthermore, mTORC1 blockade increases mouse longevity and reduces heart dysfunction associated with genetic and metabolic diseases [84]. In mouse models subjected to transverse aortic constriction, mTORC1 hyperactivation leads to pathological hypertrophy. Administering rapamycin, an mTOR inhibitor, attenuates this hypertrophic response and improves cardiac function [85]. In rat models of heart failure after myocardial infarction, rapamycin treatment enhances autophagy, reduces apoptosis, and improves cardiac remodeling by inhibiting mTORC1 signaling [86]. Using rapamycin to pharmacologically inhibit mTOR reduced age-induced cardiac inflammation and fibrosis, and even upregulates energy metabolism [87]. During myocardial ischemia, inhibiting mTORC1 promotes autophagy, which aids in cell survival. However, excessive autophagy can be detrimental in the reperfusion phase. The interplay between mTOR and autophagy-related proteins, such as Beclin1, is crucial in this context [88]. Mechanical stress activates Notch in the endocardium via the mTORC2-PKC pathway, which is important for valve formation [89]. mTORC2, through its component Rictor, inhibits the pro-apoptotic kinase MST1. Cardiac-specific deletion of Rictor leads to increased MST1 activity, resulting in cardiac dysfunction and impaired growth under pressure overload conditions [90]. mTORC2, through its component Rictor, inhibits the pro-apoptotic kinase MST1. Cardiac-specific deletion of Rictor leads to increased MST1 activity, resulting in cardiac dysfunction and impaired growth under pressure overload conditions [91].

Vascular aging and increased arterial stiffness are also key manifestations of cardiometabolic dysfunction, developing from chronic metabolic stress, inflammation, and mTOR-driven endothelial and smooth muscle remodeling. These changes reduce vascular elasticity, impair nitric oxide signaling, and promote hypertension and atherosclerosis, which links metabolic imbalance directly to cardiovascular disease progression [92]. While aging, signaling through the mTOR pathway, particularly mTORC1 (and its downstream effectors, such as S6K), becomes increasingly upregulated in vascular tissues, contributing to arterial stiffening and endothelial dysfunction. For example, in aged mice, elevated p-S6K in arteries is predominantly in the endothelium, and conditional endothelial cell-specific deletion of mTOR reverses age-related arterial stiffness and restores endothelium-dependent dilation [93]. Rapamycin-mediated pharmacologic inhibition of mTOR decreased large artery stiffness, lowered collagen accumulation in arterial walls, reduced oxidative stress and markers of cellular senescence, and even improved NO-mediated vasodilation [94]. In models of elastin deficiency, mTOR inhibition (via rapalogs) attenuates mechanosignaling, reduces medial collagen deposition, and partially restores compliance of the thoracic aorta [95]. Together, these findings establish mTOR (especially mTORC1) not only as a central mediator of vascular aging but also as a promising therapeutic target for ameliorating arterial stiffness and preserving vascular function.

## mTOR Complexity in Cardiometabolic Disease

**Fig. 2:**
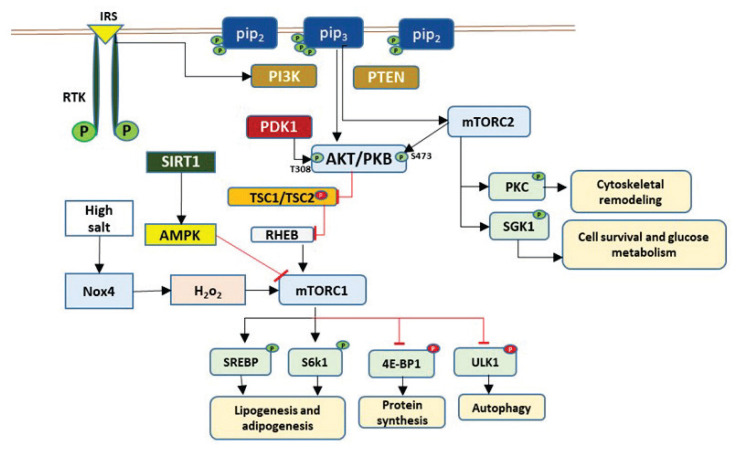
The PI3K/AKT/mTOR signaling pathway.

The mTOR regulation is extremely complex due to interrelated signaling pathways (Fig. 2), but it can be altered easily by diet, gender, severity of the disease, and age. Studies analyzing sex variations in cardiac function reveal that females frequently exhibit distinct mTORC1 activity and cardioprotective mTOR-dependent signaling compared to males, as sex hormones (estrogen, testosterone) influence cardiac mTOR signaling. In the C57BL6J mouse model, intact mTOR signalling is a prerequisite for the female cardioprotective phenotype [96]. Estrogen modulating classical estrogen receptors and G protein–coupled estrogen receptor GPER1 can activate PI3K/Akt/mTOR signalling axis, which can be anti-apoptotic and anti-autophagic [97]. Testosterone can also influence mTOR and contribute to hypertrophy in male models; inhibiting mTOR can blunt testosterone-induced cardiac changes [98]. Several reports comparing sexes/strains show tissue- and substrate-specific differences in mTORC1 readouts (p-S6, p-p70S6K, p-4E-BP1). C57BL/6J mice had increased mTORC1 activity in the liver and heart tissue of young females compared to males of the same age [99]. Kidney growth, injury responses, and fibrosis show sexual dimorphism where males and females can differ in baseline kidney mass and proliferative responses [100]. Altered mTOR signalling was observed in cystic disease and fibrosis in rat models; mTOR kinase inhibitors (PP242, etc.) improved some renal disease end-points in rat studies. However, sex-specific mTOR signalling details are less completely mapped.

## mTOR as a Diagnostic Marker in Cardiometabolic Diseases

Identifying reliable, non-invasive circulating biomarkers for mTOR pathway activation (especially mTORC1) in humans is challenging. Many downstream readout parameters are intracellular and require biopsies or cell/tissue samples. Several circulating biomarkers have been proposed to indirectly reflect mTOR pathway activation. Serum or plasma mTOR protein levels have been reported to vary with metabolic status, although they do not distinguish between active and inactive forms. Beclin-1, an autophagy-related protein inversely regulated by mTORC1, may serve as an indirect indicator of pathway activity [101]. Circulating levels of mTOR downstream effectors such as eIF4E, 4E-BP1, and S6K have been studied using genetic and proteomic approaches to infer mTOR activation, though these remain largely experimental [102]. Phosphorylated proteins like p-RPS6 and p-Akt (Ser473) show strong mechanistic links but are usually detectable only in tissues or circulating cells rather than in plasma [103]. Finally, inflammatory and metabolic markers, including IL-6, TNF-α, insulin, IGF-1, and amino acids, may indirectly signal mTOR activity through upstream pathway stimulation, but they lack specificity [104]. A review emphasizes the importance of non-invasive molecular imaging to monitor activation of the PI3K/Akt/mTOR pathway in tumor growth and chemoresistance. They describe the techniques such as [18F]FLT-PET to assess mTOR-regulated proliferation, bioluminescence or fluorescence sensors for Akt kinase activity, and FRET/BRET-based probes for real-time signaling visualization. This offers promising avenues for studying mTOR pathway activation in mTOR-mediated diseases. The development of promoter-mediated reporter techniques and circulating biomarker imaging may enhance the early detection of drug resistance mechanisms and the assessment of targeted medicines. Expanding these strategies will strengthen translational research and personalized treatment [105].

## Role of mTOR in Cardiometabolic Disorders in Dahl Rats as a Model Of Salt Hypertension

A study on Dahl SS rats shows that the mTOR complex 2 (mTORC2) plays a critical role in regulating the salt-induced blood pressure (BP) sensitivity in SS rats by regulating sodium (Na^+^) homeostasis. H_2_O_2_-mediated activation of mTORC2 has a key role in modulating Na^+^-K^+^-ATPase activity [121]. PP242, an inhibitor of the mTORC1/2 pathways, exhibits potent natriuretic actions. It completely prevents salt-induced hypertension and renal damage in SS rats by regulating renal tubular sodium and potassium transport [122]. As previously mentioned, PI3K/AKT activation is necessary for activating mTORC1; however, studies by their laboratory have highlighted an additional pathway in the kidneys that is independent of PI3K/AKT activation. In Dahl SS rats, mTORC1 activation was observed in the kidneys when they consumed a high-salt diet, which potentially triggered Nox4 (NADPH oxidase 4). Nox4 is a major source of H_2_O_2_ production that stimulates mTORC1 in the kidneys, leading thus to salt-induced hypertension and renal injury [123]. The researchers also observed that inhibition of mTORC1 with rapamycin significantly reduced albumin excretion and renal infiltration of immune cells-including CD3^+^ T lymphocytes (critical for T cell activation) and ED1^+^ macrophages (a marker of inflammation and injury in rats), in both the cortex and medulla of Dahl SS rats. This was accompanied by a moderate reduction in blood pressure [124]. Similarly, a study using PP242 treatment prevented hypertension and alleviated kidney inflammation in Dahl SS rats by inhibiting particular sodium transporters and reducing renal immune responses [125]. Sixteen weeks of high salt intake in SS rats caused significant metabolic alterations in the renal medulla and cortex. These alterations affected various pathways involved in the metabolism of organic acids, amino acids, fatty acids, and purines. In this model, high salt intake was also observed to enhance glycolysis (altering the pentose phosphate pathway) and amino acid metabolism, as well as suppress the TCA cycle (citrate synthase and aconitase) [126]. A leading study demonstrated that a high-salt diet directly induces dyslipidemia through activation of the Sterol Regulatory Element-Binding Protein (SREBP2)/Proprotein Convertase Subtilisin (PCSK9) pathway (a downstream effector of mTOR) in the liver and kidneys.

This pathway is responsible for reduced hepatic LDL receptors, resulting in hypercholesterolemia [127]. Similarly, another study showed that a high-salt diet induces hypertension and significant insulin resistance in salt-sensitive Dahl rats, which is caused by the activation of PI3K and AKT. However, supplementation of the same diet with potassium reduced metabolic defects and exerted protective effects. Furthermore, this study reveals intriguing connections between insulin sensitivity and hypertension [128].

In most organs (Fig. 2), mTOR is a critical regulator of various cellular processes, including growth, proliferation, and survival. When stimulated by growth factors such as Insulin Receptor Substrate (IRS), active Phosphoinositide 3-Kinase (PI3K) converts phosphatidyl-inositol (4,5)-bisphosphate (PIP2) into phosphatidyl-inositol (3,4,5)-trisphosphate (PIP3). This conversion facilitates the recruitment of Protein Kinase B (PKB/AKT) to the plasma membrane, where it is phosphorylated at threonine 308 (T308) by 3-Phospho-inositide-Dependent Protein Kinase-1 (PDK1) and at serine 473 (S473) by mTORC2. This leads to the full activation of AKT. Activated AKT then phosphorylates and inhibits glycogen synthase kinase 3 (GSK3) and tuberous sclerosis complex 2 (TSC2). This prevents TSC1/2 from binding to Ras homolog enriched in brain (RHEB), which allows RHEB to activate mTORC1 at the lysosomal surface. Then, activated mTORC1 phospho-rylates downstream targets, including ribosomal protein S6 kinase (S6K), eukaryotic translation initiation factor 4E-binding protein 1 (4E-BP1), and Unc-51-like autophagy-activating kinase 1 (ULK1). This regulates protein synthesis and autophagy. Additionally, active mTORC2 regulates several protein kinases, including serum/glucocorticoid-regulated kinase 1 (SGK1) and protein kinase C (PKC), as well as AKT. In schematic representations, activating phosphorylations are typically indicated by a green circle labeled “P,” and inhibitory phosphorylations are indicated by a red circle. We hypothesize that a high-salt diet may activate Nox4 (NADPH oxidase 4) in the kidneys. Nox4 is a major source of H_2_O_2_ production that stimulates mTORC1 independently of AKT. Sirtuin 1 (SIRT1), a highly conserved NAD^+^-dependent deacetylase belonging to the sirtuin family, is a post-translational regulator that modulates inflammation and maintains antioxidant potential in cells. AMP-Activated Protein Kinase (AMPK) regulates mitochondrial homeostasis and is ultimately responsible for energy supply, reactive oxygen species (ROS) generation, and intracellular calcium dynamics. SIRT1 indirectly activates AMPK by deacetylating and activating LKB1, thereby facilitating Liver Kinase B1 or Serine/Threonine Kinase 11 (LKB1)’s ability to phosphorylate and activate AMPK, which leads to increased AMPK phosphorylation and activation. Thus, they regulate each other and share many common target molecules.

## Conclusions

This review summarizes the major animal models as well as mechanisms and signaling pathways that affect mTOR in particular tissues, such as the heart, liver, and kidneys. Therefore, scrutinizing specific inhibitors of different mTOR complexes or its downstream regulators is critical for the management of CMDs. The review discusses specific mTOR complex inhibitors, including first-generation drugs like rapamycin and everolimus. We also discuss several second-generation mTOR kinase domain inhibitors, such as KU0063794 and TORKinibs (PP242 and PP30), which have been studied in cells and animals. Unlike rapamycin, these TORKinibs also inhibit mTORC2. PP242 is a far more effective mTORC1 inhibitor than rapamycin as it directly inhibits the mTOR kinase activity; unlike rapamycin, which does not block the cell’s main protein synthesis process (cap-dependent translation). Thus, these compounds may be useful when rapamycin resistance develops. The third generation of mTOR inhibitors, known as rapalinks, is currently under development [58]. Further testing of Nox4 and AKT inhibitors (downstream of mTOR) or AMPK and Sirt1 activators (which regulate mTOR) may be significant for regulating mTOR and treating certain symptoms of CMDs. Consequently, extensive research is necessary to elucidate the precise signaling pathways and the function of mTOR in the pathogenesis of cardiometabolic disorders that affect major organs. A review of these studies indicates that mTOR plays a vital role in regulating markers of cardiometabolism and inflammation, making it a potential target for treating cardiometabolic disorders.

## Figures and Tables

**Table 1:** The major animal models used to study mTOR pathways that influence cardiometabolic parameters.

Model	Disease Mimicked	Organ-specific mTOR effects	Utility	
*Raptor knockout (KO) mice*	Heart failure	Cardiac dysfunction due to mTORC1 loss	Understanding mTORC1’s cardiac functions	[106]
*Polymerase Gamma (POLG) mice*	Mitochondrial cardiomyopathy	Energy stress triggers aging and mTOR	Mitochondrial role in cardiac metabolism	[107,108]
*Transverse Aortic Constriction (TAC) model*	Pressure overload hypertrophy	Activation of mTORC1 promotes growth	Models of cardiac stress and heart failure	[109]
*Two-Kidney, One-Clip (2K1C) hypertension model*	Renovascular hypertension	mTOR drives fibrosis and hypertrophy	Cardiovascular effects of kidney dysfunction	[110]
*Ischemia-Reperfusion Injury*	Myocardial infarction	mTORC1 harmful; mTORC2 protective	Acute cardiac injury models	[111]
*Rictor knockout mice*	Stress-induced cardiac failure	Reduced Akt signaling, poor adaptation	Exploring mTORC2 in stress signaling	[90]
*Zucker Diabetic Fatty (ZDF) Rats*	Type 2 diabetes, cardiomyopathy	Hyperactive mTOR, cardiac remodeling	Metabolic interventions	[112,113]
*AMP-Activated Protein Kinase (AMPK) KO mice*	Metabolic syndrome	Disinhibited mTOR signaling	Cross-regulation between AMPK and mTOR	[114]
*Dahl Salt-Sensitive Obese (DS/Obese) Rats*	Metabolic syndrome	mTOR inhibition improves metabolic effects with potential cardiovascular side effects	Balancing metabolic benefits and cardiovascular risks of mTOR inhibition	[115]
*High-fat diet (HFD)/Cafeteria Diet Rodents*	Obesity, insulin resistance	Upregulated mTOR, metabolic remodeling	Nutrient-sensitive mTOR signaling studies	[116]
*Ob/Ob (Leptin-deficient) and (Leptin receptor-deficient) Db/Db Mice*	Obesity, insulin resistance	Overactive mTORC1 in heart and liver	Modeling the leptin pathway and obesity effects	[117,118]
*Streptozotocin (STZ)-Induced Diabetic Models*	Type 1 diabetes	Dysregulated mTOR signaling in the heart	Disease-specific therapy studies	[119]
*Aged Rodents*	Aging-related disease	Chronic mTOR activation with aging	Aging, longevity, and mTOR	[120]
